# Effects of Hemodialysis on Thiol-Disulphide Homeostasis in Critically Ill Pediatric Patients with Acute Kidney Injury

**DOI:** 10.1155/2018/1898671

**Published:** 2018-09-25

**Authors:** Ganime Ayar, Sanliay Sahin, Mutlu Uysal Yazici, Salim Neselioglu, Ozcan Erel, Umut Selda Bayrakcı

**Affiliations:** ^1^University of Health Sciences, Ankara Child Health and Diseases, Hematology Oncology Training and Research Hospital, Pediatric Intensive Care Unit, Ankara, Turkey; ^2^Hacettepe University, Faculty of Medicine, Department of Pediatric Intensive Care, Ankara, Turkey; ^3^University of Yildirim Beyazit, Ankara Atatürk Education and Research Hospital, Department of Biochemistry, Ankara, Turkey; ^4^University of Yildirim Beyazit, Department of Pediatric Nephrology, Ankara, Turkey

## Abstract

**Aim:**

To evaluate thiol/disulphide homeostasis as a new indicator of oxidative stress in AKI patients and to determine the effect of HD on antioxidant balance and oxidative stress through plasma thiols.

**Methods:**

This study was performed in patients aged between 12 months and 18 years prospectively who underwent hemodialysis due to AKI and were followed up for a year in a 22-bed tertiary pediatric intensive care unit. 20 patients and 39 controls were included.

**Results:**

No difference was present between the groups in terms of age and gender. Median values of plasma native thiol, total thiol, and percent thiol were significantly lower in AKI group both before and after dialysis when compared to control group. The median dynamic disulphide values were significantly lower in the AKI group of predialysis compared to the controls. When pre- and postdialysis values were compared, disulphide values were statistically higher after dialysis. When pre- and postdialysis native thiol, dynamic disulphide, total thiol, and percent thiol median values were compared, postdialysis values were significantly higher than the predialysis values. There was a positive correlation between albumin, total thiol, and native thiol values before dialysis in the patient group.

**Conclusion:**

AKI patients have low levels of thiol species showing the presence of oxidative stress and hemodialysis has a positive effect on thiol/disulphide balance. This new method may be an inexpensive and simple tool suitable for clinical studies and can be used in routine screening as a useful indicator to show oxidative stress.

## 1. Introduction

Acute kidney injury (AKI) is a medical problem characterized by the accumulation of breakdown products in blood as a result of the decrease in glomerular filtration rate within hours/days, which causes mortality by 50% to 80% in paediatric intensive care unit (PICU) [1, 2]. Although the pathogenesis of AKI is not clear, free oxygen radicals and antioxidant balance are involved in the pathogenesis [3–5]. Proximal tubular cells and renal medulla are especially prone to hypoxia and ischemia and poor oxygenation causes the development of tubular damage [6].

Thiol is a compound which contains sulfhydryl group (-SH) that plays a crucial role in preventing the occurrence of oxidative stress in the cells. Thiols are not only the redox tampon in the body but also a principal element of antioxidant defence system [7]. While the majority of plasma thiols mainly consist of albumin and other proteins, a minor part involves low molecular weight thiols such as cysteine, cysteinyl-glycine, glutathione, homocysteine, and *γ*-glutamylcysteine [8, 9]. Thiol groups of protein, low molecular weight compounds, cysteine residues, and the other thiol groups are oxidized by the oxidant molecules and change into reversible disulphide connective structures. The reversible disulphide connective structures can be reduced to thiol groups and therefore thiol disulphide balance is maintained. Abnormal thiol-disulphide balance levels are associated with oxidative stress [10–13]. Oxidative stress leads to oxidative destruction of lipids and other macromolecules, results in the change of the cell membrane and other cellular elements, and causes cell death and thus tissue damage and chronic diseases [9, 10]. There is a balance between oxidative stress and antioxidant defence mechanisms. There is a strong evidence of increase in morbidity of hemodialysis (HD) patients due to impairment of this balance by multiple factors [14–16]. Although HD is a commonly used renal replacement treatment, the role of dialysis in the prognosis of AKI is not fully elucidated yet [1].

Therefore, we aimed in this study to determine the thiol/disulphide balance as a new indicator of oxidative stress in AKI patients and to evaluate the effects of HD on antioxidant balance and oxidative stress through plasma thiols.

## 2. Material and Methods

This cross-sectional study was conducted in accordance with the Declaration of Helsinki and was approved by the Hospital Research Ethics Committee. Informed consent was obtained from all participants and their parents before the study. Volunteers were selected from patients who were consecutively admitted to the PICU with AKI requiring hemodialysis. Twenty patients aged between 12 months and 18 years with AKI and 39 healthy subjects were included in the study.

This study was performed at the Ankara Children's Haematology and Oncology Training and Research Hospital between June 2015 and July 2017. The thiol/disulphide homeostasis parameters were studied in the Yıldırım Beyazıt University Biochemistry Laboratory. Exclusion criteria for patients were the existence of chronic kidney failure, any hepatic disease, any form of vitamin supplementation, chromosomal and genetic syndromes, amyloidosis, severe anaemia, and malnutrition.

Physically and mentally healthy volunteers who applied to the paediatrics outpatient clinic, with no acute or chronic disease, required blood draw for any reason, and did not take any antioxidants or supplements were selected as the control group.

During the study, we used bicarbonate dialysis with polysulfone dialyzer membrane for all patients. Two millimetres of blood was collected from the hemodialysis catheter before and after dialysis from the patients who received hemodialysis during the routine blood draw. Venous blood samples of control groups were collected in tubes containing Ethylene Diamine Tetraacetic Acid (EDTA). As soon as the blood samples are collected by using vacutainer, they are centrifuged at 3600 rpm x for 10 minutes and stored in the freezer at –80°C.

### 2.1. Measurement of the Thiol-Disulphide Homeostasis Parameters

Thiol-disulphide homeostasis consists of native thiol (-SH), total thiol (-SH+-SS), disulphide (-SS), and disulphide/native thiol percent ratio (-SS/-SH %) parameters. Serum native thiol and total thiol levels were measured with a recently developed, automatic measurement method by using an automated clinical chemistry analyzer (Roche Hitachi Cobas c501 automatic analyzer, Roche Diagnostics, USA) [17]. Disulphide level and disulphide/native thiol percent ratio (index) were generated from the measured values. The half value of the difference between total thiol and native thiol amounts gave the disulphide bond amount. The index parameter was calculated based on percent ratio of disulphide and native thiol levels.

## 3. Statistical Analysis

Statistics were expressed as numbers and percentages for categorical variables for descriptive analysis and as mean, standard deviation, median, and 25^th^ percentile (Q1), 75^th^ percentile (Q3) for numerical variables. Normality of distribution was evaluated using the Shapiro–Wilk test. For two-group comparisons of non-normally distributed numerical variables, Mann-Whitney U test was employed. Wilcoxon signed rank test was used for comparison of the parameters studied before and after the dialysis. The correlation was evaluated using Spearman's correlation test. SPSS Statistics for Windows version 21.0 was used for the analysis. The statistical significance level was P<0.05.

## 4. Results

Demographics and the laboratory data of the patient group are given in Table 1. No difference was present between patient and control groups regarding age and gender. The diagnosis of patients is summarized in Table 2.  Table 3 demonstrates the comparison of thiol/disulphide homeostatic parameters in hemodialysis group. When pre- and postdialysis total thiol, native thiol, and dynamic disulphide median values were compared, postdialysis values were found to be significantly higher than the predialysis values (p<0.05). When pre- and postdialysis percent thiol values were compared, there was no statistically significant difference.

Median values of plasma native thiol, total thiol, and percent thiol were meaningfully lower in AKI group both before and after dialysis in comparison to the control group (Table 4). Total thiol was 482.00 mmol/L (IQR= 465.20-505.20) in the control group, 256.45 mmol/L before dialysis (IQR= 179.75-318-73) p=0.0001, and 324.05 mmol/L after dialysis (IQR= 276.00-410.63) p=0.0001, while native thiol was 453.40 mmol/L in the control group (IQR=431.90-464.30), 230.70 mmol/L before dialysis (IQR=182.63-293.60), and 294.45 mmol/L after dialysis (IQR=244.58-359.95), p=0.0001. There was a positive correlation between albumin, total thiol, and native thiol values before and after dialysis in the patient group (Table 5).

## 5. Discussion

The dynamic thiol/disulphide status is important in diseases where oxidative stress plays a crucial role in the pathogenesis. Thiols can undergo oxidation reaction via oxidants and form disulphide bonds. A disulphide bond is a covalent bond; the linkage is also called a SS-bond or disulphide bridge. Under conditions of oxidative stress, the oxidation of Cys residues can lead to the reversible formation of mixed disulphides between protein thiol groups and low-molecular-mass thiols. The formed disulphide bonds can again be reduced to thiol groups; thus, dynamic thiol–disulphide homeostasis is maintained [17].

Thiol can be reduced and the interventions which can increase thiol levels can stop pathological processes as a result of the disruption in the thiol/disulphide homeostasis [18]. Before this new technique, it was feasible to measure the concentrations of thiol and disulphide only in low molecular weight compounds such as cysteine, reduced glutathione (GSH), and oxidized glutathione. On the other hand, a small portion of the thiol pool of the body is composed of low molecular weight thiols (cysteine, cysteinylglycine, GSH, homocysteine, and g-glutamylcysteine); thiols of albumin and other proteins generally make up the larger fraction. Hence, thiol and disulphide concentrations measured by former methods may not show the exact thiol/disulphide status of the body. Protein thiols stand for a bigger active redox pool than glutathione [17, 19]. Thus, the homeostatic status can be completely evaluated.

Previous studies researched thiol-disulphide homeostasis in many other acute and chronic diseases [20–22]. However, there has been no study researching thiol/disulphide homeostasis in pediatric patients with AKI using a new, automated method [17]. The serum amounts of total thiol, native thiol, and dynamic disulphide connection were measured in AKI patients by a new method which objectively shows thiol-disulphide homeostasis mechanism, and given that -SS is important in the pathogenesis of oxidative stress, rates of -SS x 100 / -SH, -SS x 100 /-SS + -SH, and -SH x 100 / -SS + -SH were investigated. Our results indicated that blood thiols were significantly low in these pediatric patients with AKI. Protein thiols are the most important redox pool, and thus low redox rates show the presence of a high oxidative load. Many studies showed that the level of oxidant radicals increases in chronic renal failure (CRF) [23–26]. The increase of oxidant radical and the decrease of plasma antioxidant activity contribute to an increase in oxidative damage and development of renal complications [25]. In addition, oxidant radicals and antioxidant balance are involved in the pathogenesis of acute renal failure [3–5]; antioxidants such as superoxide dismutase have been proved to protect the person against acute renal injury related to endotoxemia and oxygen radicals causing tubule damage during ischemia [4, 5].

In patients with renal failure, the increase in oxidant radical level is multifactorial; even one of them is mentioned to be hemodialysis [23, 25]. Oxidative stress increased during hemodialysis due to numerous factors such as uremic toxicity, malnutrition, anemia, and clinical status of the patients. HD also stimulates inflammation, increasing the production of oxidant, and results in increased oxidative stress [23, 27]. It is known that when the production of oxidant agents and antioxidant defence mechanisms is imbalanced, an important source of morbidity arises [28].

In our results, there were significantly higher postdialysis values of native thiol, dynamic disulphide, and total thiol compared to predialysis values. There are similar studies in the literature showing the effect of hemodialysis on antioxidant homeostasis [10, 24, 26, 29]. In a study conducted by Colombo et al., it was found that lower plasma levels of total thiols in patients with end-stage kidney disease compared to the healthy controls were the same as those of the healthy persons' after dialysis, and they attributed this result to hemodialysis being the first session [26]. Although in our results postdialysis plasma thiol levels were not the same as those of the healthy group, these values have increased compared to the predialysis. As in our study, hemodialysis sessions may restore plasma thiol redox status in the acute period, but antioxidant capacity may be insufficient as the status becomes chronic. There are different studies which demonstrated the total serum antioxidant levels in chronic hemodialyzed patients compared to the healthy group [30]. Furthermore, some studies have found higher total serum antioxidant levels at predialysis [31, 32], and some have found a significant decrease in antioxidants after dialysis [32]. Different results might be caused by different tests used for measurement of antioxidants and the results might be affected by many patient-related factors [7]. However, we consider our study important since the plasma thiols are the largest antioxidant components of serum.

The difference between the patient and control groups in our study was statistically significant. Lower plasma thiol levels in predialysis and postdialysis, compared to the control group, were consistent with the literature [16, 33, 34], suggesting that these patients are susceptible to oxidative damage. On the other hand, postdialysis thiols values were significantly higher than those of the predialysis, so that hemodialysis may have a positive effect on the oxidative balance in patients with AKI.

Among other antioxidant molecules, albumin was also low in the study group, and lower levels of albumin were found in predialysis patients, when compared to the postdialysis which suggests us that there was a positive correlation between albumin and thiol values.

Our study has a few limitations. The most significant one of these was the limited number of patients. And we ignored the number of patients during the dialysis session while obtaining blood samples. The single centred study was another limitation of this study.

## 6. Conclusion

This study showed that HD may affect the thiol/disulphide homeostasis. According to the findings, AKI patients have low levels of thiol species showing the presence of oxidative stress; and hemodialysis has a positive effect on thiol/disulphide homeostasis. Although further studies with larger sample groups including recurring dialysis sessions are required, it must be underlined that this new method may be an inexpensive and simple instrument suitable for medical studies that can be applied to routine screening as a useful indicator to show thiol-specific oxidative stress.

## Figures and Tables

**Table 1:** Demographics and laboratory data.

**Parameters**	** Case Group ** ** (n=20)**	** Control Group ** ** (n=39) **	**p**
**Sex (females/males)**	11/9	22/17	0.918
**Age, years** **∗**	12 (7.5-15.8)	10 (7.0-13.0)	0.265
**Hemoglobin (g/dL) pre-dialysis** **∗**	9.2(7.9-10.4)	11.8 (10.7-12.1)	<0.001
**Albumin (g/dL) pre-dialysis** **∗**	3.5 (3.0-3.9)	4.2 (3.9-4.4)	<0.001
**Creatinine (mg/dL) pre-dialysis** **∗**	3.3 (2.2-5.7)	0.4 (0.3-0.5)	<0.001

∗Median (Q1-Q3).

**Table 2:** Diagnosis distribution in patient group.

	**Case Group ** ** (n=20)**
**Drug Intoxication**	4
**Metabolic Disorder**	4
**Hypoxic ATN**	3
**Hemolytic Uremic Syndrome**	3
**Sepsis**	2
**Kardiyomyopati (Heart Failure)**	1
**Nefritic Syndrome**	1
**Hydronephrozis (Urinary tract obstruction) **	1
**Polycystic Kidney Disease**	1

**Table 3:** Comparison of thiol/disulphide homeostatic parameters in hemodialysis group.

	**Pre-dialysis**	**Post-dialysis**	**p**
	Median (Q1-Q3)	Median (Q1-Q3)	
Native thiol (*µ*mol/L)	230.70 (182.63-293.60)	294.45 (244.58-359.95)	** 0.003** **∗**
Total thiol (*µ*mol/L)	256.45 (179.75-318-73)	324.05 (276.00-410.63)	**0.003** **∗**
Disulphide (*µ*mol/L)	13.50 (10.56-17.31)	15.35 (12.92-21.15)	**0.021** **∗**
Disulphide/native thiol (%)	5.62 (4.44-7.88)	6.15 (4.16-7.77)	0.747
Disulphide/total thiol (%)	5.08 (4.03-6.96)	5.47 (3.84-6.72)	0.778
Native thiol/total thiol (%)	89.00 (85.50-91.83)	89.70 (86.54-92.46)	0.841

^**∗**^p<0.05 was considered significant for statistical analyses (Wilcoxon signed rank test).

**Table 4:** Comparison of thiol/disulphide homeostatic parameters between control and hemodialysis group.

****	**Control** Median (Q1-Q3)	**P** **Pre-dialysis vs. Control**	**P** **Post-dialysis vs.** **Control**
Native thiol (*µ*mol/L)	453.40 (431.90-464.30)	**0.0001** **∗**	**0.0001** **∗**
Total thiol (*µ*mol/L)	482.00 (465.20-505.20)	**0.0001** **∗**	**0.0001** **∗**
Disulphide (*µ*mol/L)	19.55 (14.95-21.70)	**0.0001** **∗**	0.113
Disulphide/native thiol (%)	4.15 (3.26-4.82)	**0.001** **∗**	** 0.001** **∗**
Disulphide/total thiol (%)	3.83 (3.06-4.40)	**0.002** **∗**	** 0.001** **∗**
Native thiol/total thiol (%)	92.33 (91.20-93.87)	**0.001** **∗**	** 0.002** **∗**

**∗**p<0.01 was considered significant for statistical analyses (Mann-Whitney U test).

**Table 5:** Correlations between thiol parameters and other parameters.

**Pre-dialysis**	Native thiol		Total thiol		Disulphide	

	r	p	r	p	r	p

Age	-0.291	0.214	-0.303	0.195	0.124	0.602
Hemoglobin	-0.333	0.152	-0.337	0.146	-0.288	0.219
Albumin	0.646	**0.002** **∗**	0.643	**0.002** **∗**	0.192	0.418
Creatinine	0.087	0.715	0.113	0.636	0.361	0.118

**Post-dialysis**	Native thiol		Total thiol		Disulphide	

	r	p	r	p	r	p

Age	-0.132	0.580	-0.110	0.643	-0.203	0.391
Hemoglobin	-0.342	0.140	-0.339	0.144	0.075	0.755
Albumin	0.445	**0.049** **∗**	0.464	**0.039** **∗**	0.366	0.112
Creatinine	0.057	0.811	0.042	0.860	-0.299	0.200

**Control**	Native thiol		Total thiol		Disulphide	

	r	p	r	p	r	p

Age	0.106	0.519	0.090	0.584	-0.049	0.769
Hemoglobin	0.104	0.529	0.066	0.691	0.071	0.669
Albumin	0.033	0.840	0.013	0.939	0.033	0.841
Creatinine	-0.256	0.116	-0.205	0.211	-0.041	0.804

∗p<0.05 was considered significant for statistical analyses (Spearman's correlation test).

## Data Availability

The data used to support the findings of this study are available from the corresponding author upon request.
